# QuanDB: a quantum chemical property database towards enhancing 3D molecular representation learning

**DOI:** 10.1186/s13321-024-00843-y

**Published:** 2024-04-29

**Authors:** Zhijiang Yang, Tengxin Huang, Li Pan, Jingjing Wang, Liangliang Wang, Junjie Ding, Junhua Xiao

**Affiliations:** State Key Laboratory of NBC Protection for Civilian, Beijing, People’s Republic of China

**Keywords:** Database, Quantum chemical properties, Cheminformatics, Machine learning

## Abstract

Previous studies have shown that the three-dimensional (3D) geometric and electronic structure of molecules play a crucial role in determining their key properties and intermolecular interactions. Therefore, it is necessary to establish a quantum chemical (QC) property database containing the most stable 3D geometric conformations and electronic structures of molecules. In this study, a high-quality QC property database, called QuanDB, was developed, which included structurally diverse molecular entities and featured a user-friendly interface. Currently, QuanDB contains 154,610 compounds sourced from public databases and scientific literature, with 10,125 scaffolds. The elemental composition comprises nine elements: H, C, O, N, P, S, F, Cl, and Br. For each molecule, QuanDB provides 53 global and 5 local QC properties and the most stable 3D conformation. These properties are divided into three categories: geometric structure, electronic structure, and thermodynamics. Geometric structure optimization and single point energy calculation at the theoretical level of B3LYP-D3(BJ)/6-311G(d)/SMD/water and B3LYP-D3(BJ)/def2-TZVP/SMD/water, respectively, were applied to ensure highly accurate calculations of QC properties, with the computational cost exceeding 10^7^ core-hours. QuanDB provides high-value geometric and electronic structure information for use in molecular representation models, which are critical for machine-learning-based molecular design, thereby contributing to a comprehensive description of the chemical compound space. As a new high-quality dataset for QC properties, QuanDB is expected to become a benchmark tool for the training and optimization of machine learning models, thus further advancing the development of novel drugs and materials. QuanDB is freely available, without registration, at https://quandb.cmdrg.com/.

## Scientific contribution

- The QuanDB database contains comprehensive quantum chemical properties of diverse organic molecular entities, and all data have been rigorously pretreated and manually cleaned to ensure high accuracy.
- By utilizing the quantum chemical properties provided by QuanDB, relevant three-dimensional (3D) electronic structural information can be included in comprehensive molecular representation models to facilitate drug and material design.
- Compared to other similar databases, QuanDB covers a broader space of chemical compounds, adopts a higher level of theoretical calculations, and offers a user-friendly interface.

## Graphical abstract

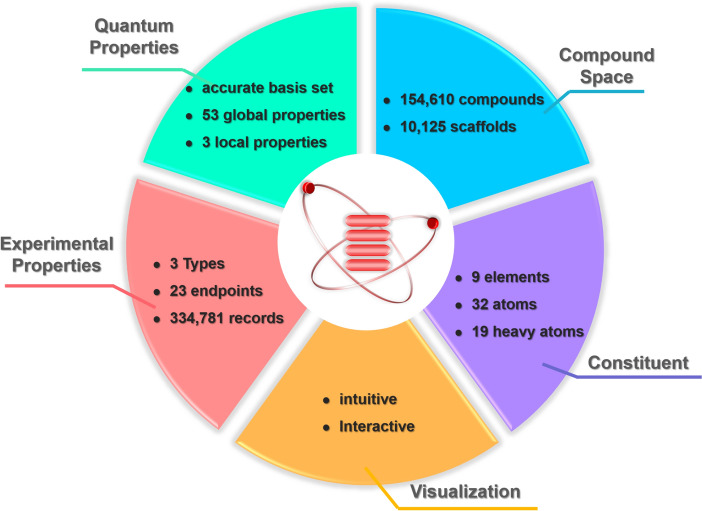

## Introduction

Currently, the fundamental assumption of an AI-assisted molecular (drug or material) design is that “structurally similar molecules have similar properties.” A comprehensive molecular representation is crucial for facilitating the discovery of novel molecules [1]. Generally, molecular representations with stronger discriminative ability tend to demonstrate superior performance in downstream molecular design tasks [2–5]. Traditional molecular descriptors require manual feature engineering, making it difficult to comprehensively represent molecules without expert knowledge [6]. Consequently, data-driven representation models are increasingly used to extract unbiased features from molecules [7–10]. Additionally, as the relationships between the molecular structure and physicochemical (PC) properties, reactivity, and bioactivity are becoming better understood, researchers are gradually incorporating features that can include the three-dimensional (3D) conformation of molecules in representation models [11–14]. The electronic and structural parameters of stable 3D conformations are of particular interest because they critically affect several crucial properties of molecules in 3D space, such as their reactivity, strong electrostatic interactions, and chemical adsorption. Density functional theory (DFT) remains the most reliable and accurate method for obtaining the electronic structure information of the most stable 3D molecular conformations, which can be reflected by quantum chemical (QC) properties [15–18]. By incorporating QC properties into the training phase of the molecular representation models, their ability to represent the electronic structural space can be effectively enhanced, thereby improving the performance of downstream tasks, such as predicting molecular properties [12, 19]. Therefore, the construction of a DFT-based QC property database for small organic molecules is of great significance for the virtual evaluation, screening, and reverse design of novel molecules.

QC property databases aim to comprehensively represent the electronic structural information of the most stable 3D molecular conformations using a broad set of QC properties [20–22]. The QM9 database is currently the most extensively used and authoritative source of QC properties [22]. It comprises data for 134,000 molecules taken from the GDB-17 database [23]. Presently, QM9 plays a crucial role as a benchmark dataset for evaluating molecular representation models [2, 3] and producing 3D molecular representations [24–29]. However, the QM9 dataset has several limitations. First, the geometric structural optimization in QM9 is performed at the B3LYP/6-31G(2df,p) theoretical level, which allows for potential improvements in the calculation accuracy [30]. Second, to reduce computational complexity, QM9 restricts the number of heavy atoms to a maximum of 9 and contains only 5 elements: H, C, O, N, and F. This limitation severely restricts the representation of diverse molecular structures and chemical compound spaces. Despite these limitations, the simple molecular structures in the QM9 dataset have yielded excellent predictive results when used as input data for current deep-learning models [3, 12], some of which achieved prediction errors close to zero [4]. Additionally, the molecules in the QM series datasets are computed and thus deviate to some extent from real materials. Finally, QM9 lacks a user-friendly visualization interface, which makes it difficult for researchers outside the field to take full advantage of its utility. In conclusion, it is imperative to develop a new database of high-quality QC properties that contains real compounds, has broad coverage of the chemical space, and provides a user-friendly interface.

To address the need for a new database, this study aimed to develop a new high-quality QC property database, which comprises diverse labeled compounds with more comprehensive QC properties than previous databases and a user-friendly interface. It can not only further enrich and supplement high-value molecular structure representation information, but also provide a benchmark for the training and optimization of machine learning models, thereby facilitating the design and development of novel materials and drugs.

## Construction and content

### Data collection and curation

**Fig. 1:**
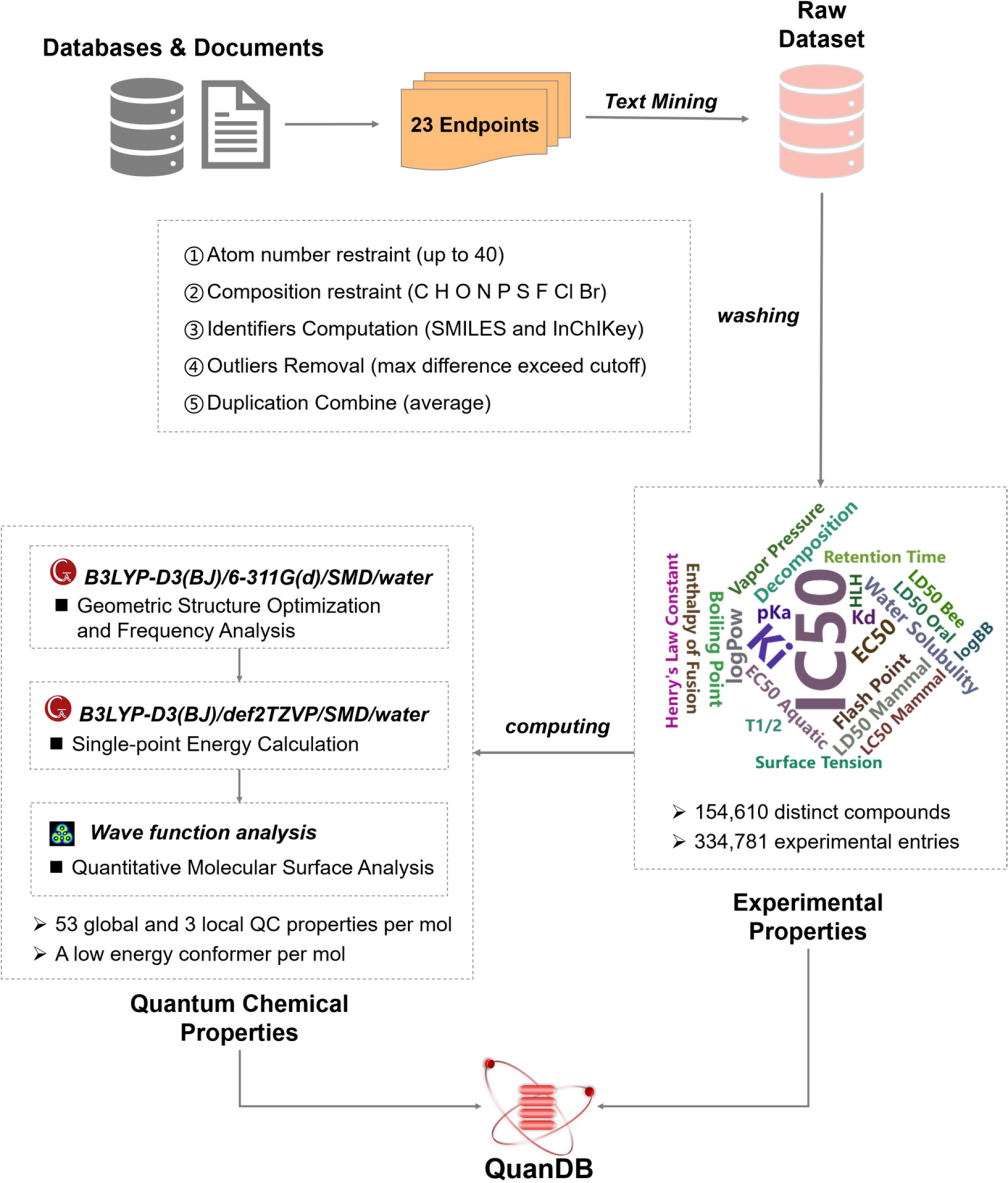
Data collection and cleaning process in QuanDB. The 23 proposed endpoint properties are listed in Table 1. Each molecule in QuanDB contains 53 global and 5 local QC properties, as well as the lowest energy conformation

First, to identify the target molecular entity in the database based on the research requirements, 23 endpoints were defined, covering three categories: bioactivity, toxicity, and PC properties (Table 1). We used a semi-automatic text-mining method to collect experimental data from databases such as OCHEM [31], PubChem [32], and DrugBank [33], which include the one of above 23 endpoints for compounds and annotated the literature sources. A good database should cover the largest possible chemical space. However, since the computational time is exponentially related to the number of atoms, the maximum learning space was limited by the computational resources available to our research group. Therefore, we restricted the range of elements to C, H, O, N, P, S, F, Cl, and Br, with a maximum of 40. Based on these constraints, we removed small molecules from the original data that exceeded these limits, along with their corresponding experimental values. Considering that different experimental data can be obtained for the same molecule and endpoint (e.g., due to variations in the experimental conditions), we performed data deduplication and cleaning steps. The cleaning strategy was as follows: if the maximum ratio of logarithmic values for duplicate molecule entries exceeded 1.17 (log_10_ 15), all data were deleted; otherwise, the mean value was used as the final experimental value. Because the geometric structure of a molecule significantly affects its quantifiable properties at the microscopic level, molecules with different conformations were treated as different entities. Finally, we calculated the basic properties such as relative molecular mass, Canonical SMILES, InChI, and InChIKey for each molecule and annotated them. Finally, we obtained 154,610 molecule entities with 334,781 property data entries for 23 endpoints. The overall data-cleaning process is shown in Fig. 1.

**Table 1:** Endpoint properties and corresponding categories in QuanDB

Endpoint	Type	Endpoint	Type
IC_50_	Bioactivity	T_1/2_	PC
EC_50_	Bioactivity	Vapor pressure	PC
*K*_i_	Bioactivity	Water solubility	PC
*K*_d_	Bioactivity	log BB	PC
Boiling point	PC	Log Pow	PC
Decomposition	PC	p*K*_a_	PC
Enthalpy of fusion	PC	EC_50_ aquatic	Toxicity
Flash point	PC	LC_50_ mammal	Toxicity
HLH	PC	LD_50_ bee	Toxicity
Henry's Law Constant	PC	LD_50_ mammal	Toxicity
Retention time	PC	LD_50_ oral	Toxicity
Surface tension	PC	—	—

### Calculation and extraction of QC properties

Based on the tradeoff between the calculation accuracy and computational time, the basis set with the highest accuracy obtainable using our limited computing resources was chosen. For geometric structural optimization, which has low conformational sensitivity to the basis set and is highly time-consuming, we chose a common 3-zeta basis set, viz., 6-311G(d) [34]. For the single-point energy calculation, which is sensitive to the basis set, we chose a higher-level 3-zeta basis set (def2-TZVP) [35].

The calculation of the QC properties involves the following three steps. (1) The GMMX3.0 module in GaussView6 [36] is used to search for molecular conformations. The lowest-energy conformation is then subjected to geometric structure optimization and frequency analysis using Gaussian16 [37] at the B3LYP-D3(BJ)/6-311G(d)/SMD/water [38] theoretical level. After obtaining the lowest-energy conformation without imaginary frequencies, the single-point energy calculation is performed at the B3LYP-D3(BJ)/def2-TZVP/SMD/water [38] theoretical level. (2) The Gaussian16 wavefunction file (.chk) is analyzed using Multiwfn software to obtain a.txt file containing the molecular electrostatic surface properties. (3) QC properties are extracted automatically in batches using internal scripts. In total, we obtained 53 global and 5 local QC properties, as well as the lowest-energy conformation for each molecule (Fig. 1). Therefore, the QC properties in QuanDB are derived from three sources: (i) properties obtained from the geometric structural optimization and frequency analysis; (ii) properties calculated from the single-point energy of the lowest-energy conformation obtained in (i); and (iii) properties obtained from quantitative surface analysis of the wavefunction file using Multiwfn software [39].

### Online database implementation

The backend service of the QuanDB database was built using the Python web framework FastAPI, whereas the frontend pages were developed using Vue 3.0 [40]. The entire database follows a backend/frontend separation, essentially implementing the MVVM pattern. All data are stored and managed using MySQL software. For molecular visualization, the RDKit toolkit [41] is used to generate two-dimensional (2D) graphs and 3D structures are displayed using 3Dmol.js [42]. All visualizations in QuanDB are implemented using ECharts [43]. QuanDB has undergone comprehensive testing to ensure functionality across multiple operating systems and web browsers.

### Current database content and statistics

QuanDB is a comprehensive and user-oriented QC property database that is proposed as a high-quality benchmark for QC properties. QuanDB can be used to represent the most stable 3D conformations and electronic structures of small organic molecules, such as drugs and other materials. In turn, it could play an important role in a range of downstream tasks like property prediction, molecule generation, and inverse molecule design.

**Fig. 2:**
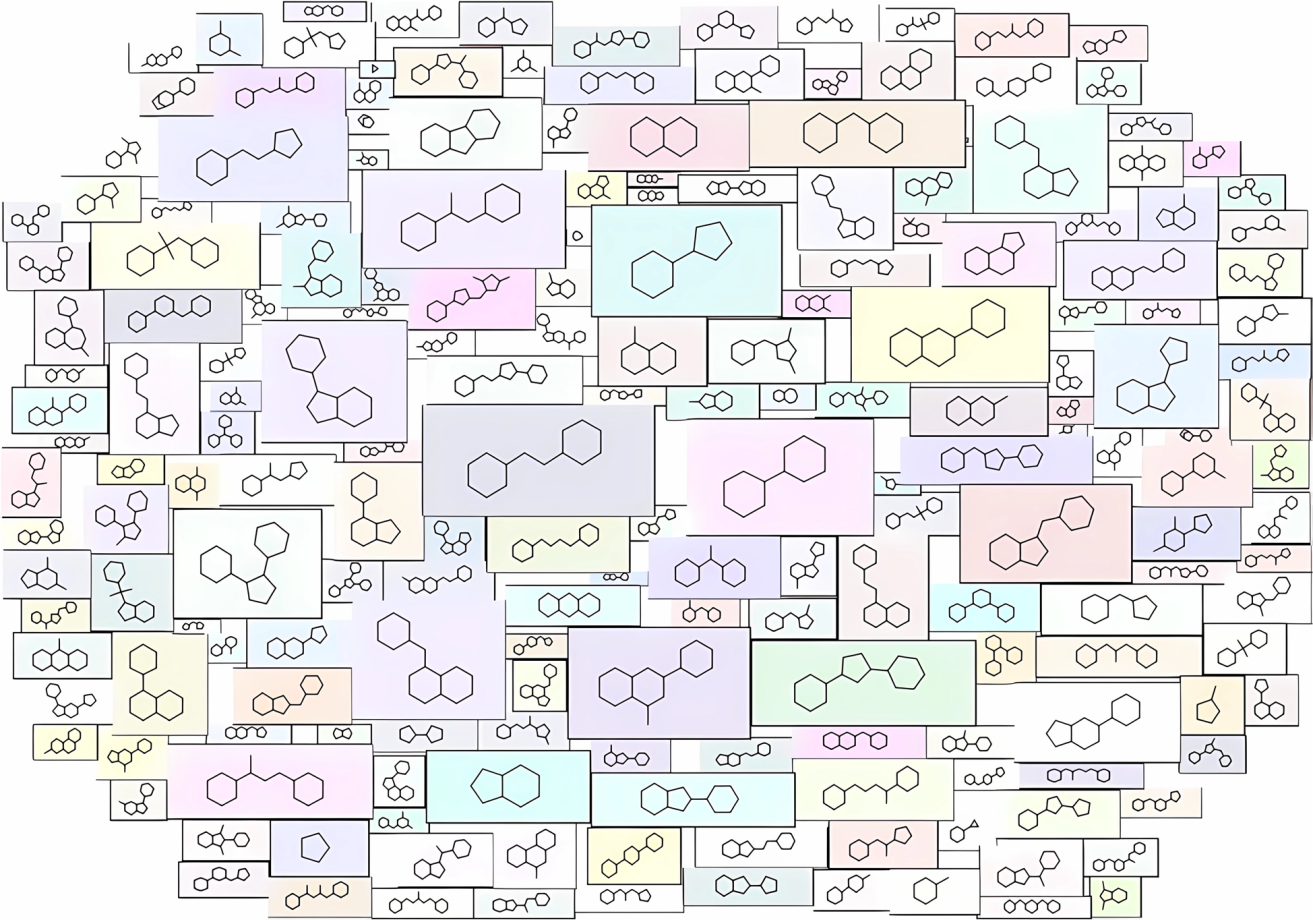
Top 200 scaffolds cloud diagrams in QuanDB, excluding the cyclohexane. The size of the scaffolds layer is attributed to the corresponding frequency

The primary objective of most chemical databases is to explore a wide chemical compound space. To cover the chemical space of practically relevant compounds as much as possible while minimizing computational complexity, QuanDB restricts the elemental composition to H, C, O, N, P, S, F, Cl, and Br and loosens the upper limit for the total number of atoms to 40. In total, QuanDB includes 154,610 molecular entities and 10,125 scaffolds. On average, each scaffold covers 14 molecules, and more than 85% of scaffolds contain fewer than 10 molecules. Approximately 46% of the scaffolds are distinct. A cloud diagram of the top 200 scaffolds is shown in Fig. 2, as implemented using the Scopy toolkit [44].

**Fig. 3:**
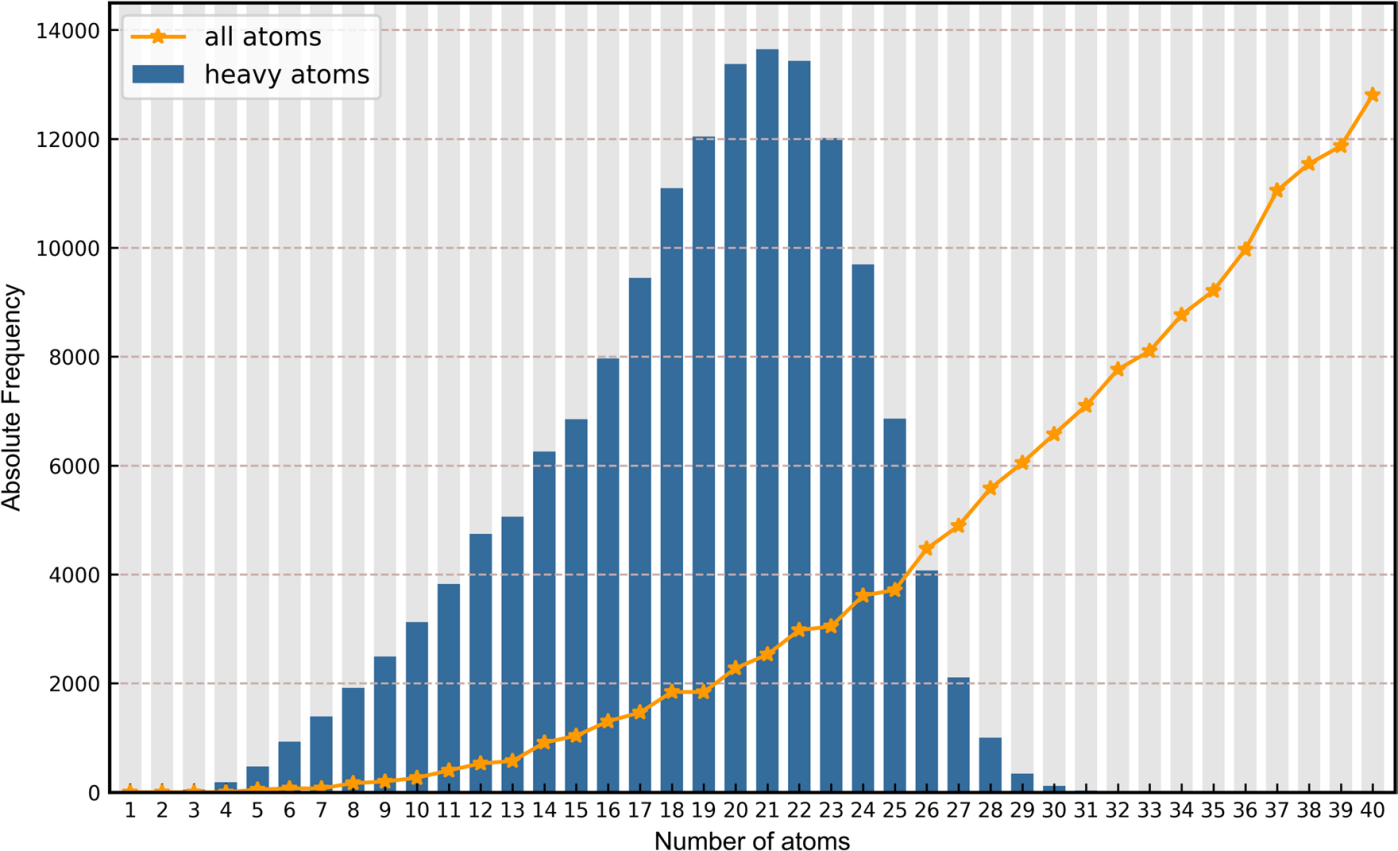
Frequency distribution of atoms in QuanDB, including heavy atoms (blue bar chart) and all atoms (orange line graph)

In terms of molecular composition, the distribution of total atoms and heavy atoms in QuanDB can is shown be seen in Fig. 3. On an average, the molecules in QuanDB contain 19 heavy atoms, whereas the most widely used QC property database (QM9the most widely used QC property database) has a limit, QM9, contains a maximum of 9 heavy atoms. In terms of heteroatoms, the three most frequently occurring elements are N, O, and F. Overall, compared with other popular QC property benchmarks, QuanDB provides a better means to test and evaluate the representation and generalization ability of models, and presents a greater challenge to their goodness of fit.

**Fig. 4:**
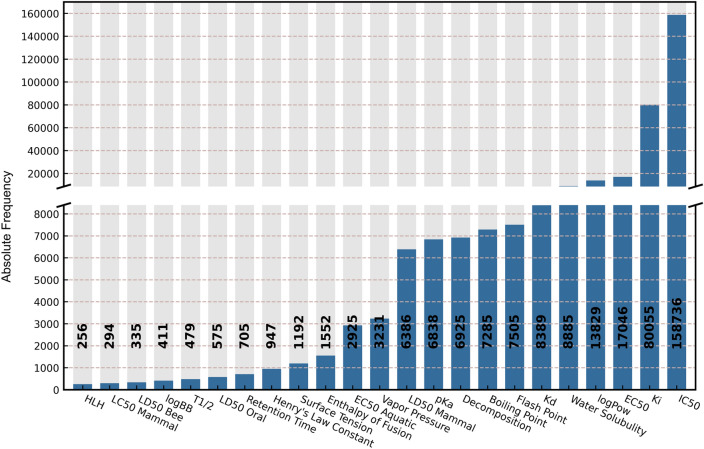
Experimental endpoints in QuanDB, along with their frequency distributions

Another advantage of QuanDB is that the molecular entities are accompanied by experimental data. Currently, QuanDB contains 334,781 rigorously validated experimental data points for 23 endpoint properties (Fig. 4). Among these properties, bioactivity, toxicity, and PC properties account for 79%, 2%, and 19%, respectively. Among them, IC_50_ has the highest number of entries (136,746), accounting for 47% of the entire dataset. For this subset of data, we annotated the target of compound action and the PubMed ID. Overall, QuanDB provides a high-quality, standardized dataset for drug and material design.

QuanDB provides 53 global QC properties (e.g., zero-point energy) and 5 local QC properties (four atomic charges and one chemical bond order) for each molecule. These QC properties were categorized into three types based on their representative level: geometric, electronic, and thermodynamic properties. Additionally, QuanDB provides the lowest-energy conformation for each molecule, thus serving as a standard dataset for conformer-generation research. The high-quality experimental data and accurate QC properties provided by QuanDB highlight its potential as a benchmark dataset for evaluating model performance. In addition, the electronic structural information is expected to be useful for molecular representation learning, thus enhancing model representation capabilities. A complete list of the 58 QC properties in QuanDB is presented in Table 2.

**Table 2:** QC properties in QuanDB and their abbreviations

Level	Source	Type	Endpoint	Abbr
Global	OptFreq	Thermo-dynamics	Zero-point energy	ZPE
			Total energy	E_opt_
			Total energy under 0 K	E_0k_
			Internal energy	U
			Thermal enthalpy	H
			Entropy under	S
			Gibbs free energy	G
			Heat capacity	C_v_
			Thermal correction to internal energy	U_corre_
			Thermal correction to thermal enthalpy	H_corre_
			Thermal correction to Gibbs free energy	G_corre_
		Electronic structure	Energy of HOMO	E_homoOpt_
			Energy of LUMO	E_lumoOpt_
			Energy between EhomoOpt and ElumoOpt	E_gapOpt_
			Electronic spatial extent	ESE_opt_
			Dipole moment	μ_opt_
			Quadrupole moment in traceless format	Θ_opt_
			Isotropic polarizability	α_opt_
	SPE. Cal	Geometric structure	Molar volume by Monte Carlo algorithm	Volume_MC_
		Thermo-dynamics	Total energy	E
		Electronic structure	Energy of HOMO	E_HOMO_
			Energy of LUMO	E_LUMO_
			Energy between EHOMO and ELUMO	E_gap_
			Electronic spatial extent	ESE
			Dipole moment	μ
			Quadrupole moment in traceless format	Θ
			Maximum electrostatic potential charge	ESPC_max_
			Maximum Hirshfeld charge	Hirshfeld_max_
			Maximum electrostatic potential charge	CM5_max_
			Maximum natural population analysis atom charge	NPA_max_
			Minimum electrostatic potential charge	ESPC_min_
			Minimum Hirshfeld charge	Hirshfeld_min_
			Minimum electrostatic potential charge	CM5_min_
			Minimum natural population analysis atom charge	NPA_min_
	QMSA	Geometric structure	Volume by improved marching Tetrahedra algorithm	Volume_IMT_
			Estimated density according to mass and volume	Density
			Overall electrostatic potential surface area	SA
		Electronic structure	Positive electrostatic potential surface area	SA^+^
			Negative surface electrostatic potential area	SA^−^
			Nonpolar electrostatic potential surface area	SA_nonpolar_
			Polar electrostatic potential surface area	SA_polar_
			Average of negative electrostatic potential	ESP_μ_
			Average of positive electrostatic potential	ESP_μ_^+^
			Average of negative electrostatic potential	ESP_μ_^−^
			Variance of overall electrostatic potential	ESP_σ_
			Variance of positive electrostatic potential	ESP_σ_^+^
			Variance of negative electrostatic potential	ESP_σ_^−^
			Maximum of overall electrostatic potential	ESP_max_
			Minimal of overall electrostatic potential	ESP_min_
			Balance of charges	ν
			Product of ν and σ2	νESP_σ_
			Internal charge separation	Pi
			Molecular polarity index	MPI
Local	SPE. Cal	Electronic structure	Electrostatic potential charge	ESPC
			Hirshfeld charge	Hirshfeld
			CM5 charge	CM5
			Natural population analysis atom charge	NPA
			Wiberg bond order	Wiberg

Source: computational source of quantitative properties

OptFreq: geometric structure optimization and frequency analysis using the B3LYP-D3(BJ)/6-311G(d)/SMD/water method

SPE. Cal.: single-point energy calculation using the B3LYP-D3(BJ)/def2-TZVP/SMD/water level of theory

QMSA: quantitative molecular surface analysis conducted using Multiwfn software

## Utility and discussion

### Web design and interface

QuanDB offers researchers a user-friendly interface to facilitate the access and use of its extensive data. The QuanDB database is available at https://quandb.cmdrg.com. The search box at the top enables users to input the SMILES of a molecule or draw its structure. The ‘Browse’ option in the navigation bar allows users to explore the entire dataset, whereas the ‘Download’ feature provides multiple methods for data retrieval. Further assistance can be found in the ‘Help’ section.

#### Data browsing

By default, the browsing interface of QuanDB displays all molecular structures in the QuanDB identifier order. On the left-hand side of the browsing page, the filters are designed based on the endpoints of the experimental properties, and users can filter compounds that contain the desired endpoints based on the requirements of their specific application. To select multiple endpoints, the database uses the “OR” logical operator for processing. In addition, the number of molecules displayed per page can be controlled using a selector at the top of the page. Finally, clicking on a compound card in the browsing interface opens the corresponding page with a detailed description, including the basic chemical properties and quantitative descriptors of the molecule.

#### Data searching

The search box on the right-hand side banner on any QuanDB page provides a quick search function. Users can enter the SMILES code or draw the structure of the query molecule using tools provided by the database in the search box, and then click the search icon to initiate the search. After a few seconds, the user is redirected to the search results page. The search interface page is similar to the browsing interface, but the molecules are sorted in the descending order of similarity to the query molecule (based on the Tanimoto Index and 1024-bit ECFP4 Fingerprints), and the query structure is fixed on the right-hand side of the page. Similarly, users can filter the experimental data and perform other operations. Clicking on a compound card redirects the user to the corresponding detailed information page.

#### Data retrieval

**Fig. 5:**
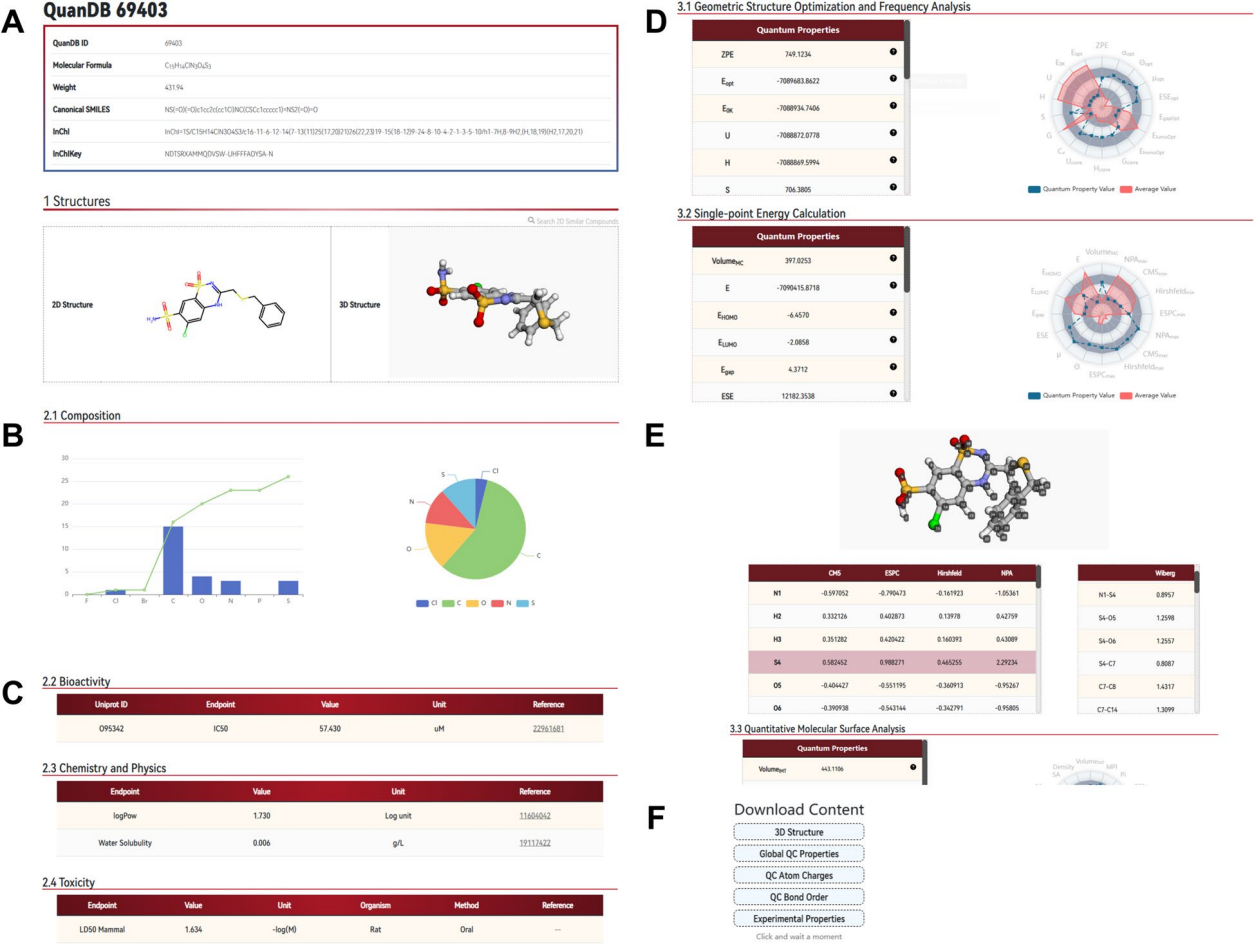
Molecular information page in QuanDB: **A** Basic information of the molecule, including common molecular identifiers and 2D and 3D structures (QuanDB 69403); **B** Composition of the molecule, with a bar chart on the left showing the frequency distribution of each element, a line chart showing the cumulative distribution of heavy atoms, and a pie chart on the right showing the proportion of each heavy atom; **C** Three major categories of experimental data for the molecule; **D** Display of global QC properties of the molecule, with a radar chart on the right showing the distribution of properties relative to the overall database mean; **E** Interactive table for displaying local QC properties design; **F** Download area

The information page for a selected molecule consists of three sections: basic information, experimental and QC property data, and corresponding charts of the properties. As shown in Fig. 5A, the Basic Information section provides the QuanDB ID and other characteristics, including the molecular formula, molecular weight, SMILES, InChI, and InChIKey. To provide a high-quality dataset for 3D molecular representation learning, QuanDB provides two-dimensional structures and the lowest-energy conformations obtained by energy optimization using Gaussian 16 (based on B3LYP-D3(BJ)/6-311G(d)/SMD/water). Additionally, upon clicking “Search 2D Similar Compounds” on the structure card, a search is performed using the current molecule as the query structure and the user is redirected to the search results page. Next, in the Experimental Data section, the elemental composition and distribution are presented in the form of bar and pie charts, respectively, to provide an approximate representation of the chemical space of the molecule. The green line in the bar chart represents the cumulative number of atoms (Fig. 5B). Below the elemental composition diagram, experimental data related to bioactivity, PC properties, and toxicity are displayed separately (Fig. 5C). Each record is processed using the method mentioned in the “Data collection and curation” section. If available, links to relevant literature are provided for user reference. For bioactivity data, QuanDB not only indicates the endpoint (e.g., IC_50_), but also provides the corresponding UniProt ID for the target. In the case of toxicity data, information on the organism and administration route are provided to clarify the nature of the endpoint as much as possible.

Finally, the QC properties in the QuanDB database are divided into three sections based on the method of acquisition: “Geometric Structure Optimization and Frequency Analysis,” “Single-point Energy Calculation,” and “Quantitative Molecular Surface Analysis.” The calculated values of the corresponding properties are displayed in the tables on the left side of each section. The radar chart on the right shows the distribution of molecular properties in the overall database. The blue line in the chart represents the properties of the molecule, whereas the red area represents the geometric mean value of all data in the database for that endpoint (Fig. 5D). Additionally, in the “Single-point Energy Calculation” section, five types of local QC properties are provided, i.e., four types of atomic charges and one chemical bond order. To present the results intuitively, QuanDB offers an interactive table to enable the user to hover over specific atoms or chemical bonds, which are then highlighted in the structure above (Fig. 5E). Furthermore, the download section on the right side of the page allows users to download desired information based on their requirements (Fig. 5F).

### Downloads and updates

Users can download the experimental properties, QC properties, and the lowest-energy conformations of molecules from the database without the need to log in or register. The dataset is divided into multiple subsets based on the 23 experimental endpoints as most applications of the database are expected to be focused on specific molecular endpoints. We hope that these datasets will assist researchers in exploring QC properties and establishing more comprehensive molecular representation models. In the future, we will continue to maintain and update the database; the QC properties and experimental data will be updated every 6 months as new molecules are computed and processed. In addition, we plan to perform calculations for more complex molecules.

## Conclusions

Many key PC properties and biological activities of molecules are closely related to their 3D geometric and electronic structures. Therefore, the construction of a high-quality property database is very important for facilitating the further development of molecular representation models. Considering the limitations of existing available public QC property databases, we developed QuanDB as a more targeted and higher-quality QC database. Currently, the QuanDB contains 154,610 molecular entities, 10,125 scaffolds and 334,781 experimental labels. For each molecule, 53 global and 5 local QC properties, as well as the lowest-energy conformation are provided, with a total computational cost of more than 10^7^ core-hours. The advantages of this database compared to existing ones are as follows: (i) All the molecular entities are labeled compounds, and the experimental data cover 23 experimental property endpoints. (ii) The molecular structure types are more diverse, covering a wider chemical compound space, including nine elements (C, H, O, N, P, S, F, Cl, and Br), and allowing up to 40 atoms in each molecule. (iii) More comprehensive QC properties are provided, and the automated batch calculations and extraction of QC properties are realized by combining Gaussian16, Multiwfn, and in-house scripts. (iv) The database has a user-friendly interface, with intuitive and interactive features. The 23 endpoints are categorized into three major classes: bioactivity, toxicity, and PC properties. Users can download the entire dataset or specific subsets according to their needs.

In general, QuanDB is a high-value QC database supported by current computing power. We expect that QuanDB will become a valuable tool for enhancing the representation capability of molecular representation models, while providing a new benchmark for researchers to develop QC property prediction models. This will ultimately contribute to advancement in molecular design research.

## Data Availability

The whole data in QuanDB can be retrieved via https://quandb.cmdrg.com/#/download without any requests, and the calculation scripts used in this work can be obtained through https://github.com/kotori-y/scripts_4_quandb. Besides, for developers we also provide a REST API for accessing: https://quandb.cmdrg.com/api/docs.

## Abbreviations

3D: Three-dimensional
DFT: Density functional theory
PC: Physicochemical
QC: Quantum chemical
