# Correction: QuanDB: a quantum chemical property database towards enhancing 3D molecular representation learning

**DOI:** 10.1186/s13321-024-00864-7

**Published:** 2024-06-11

**Authors:** Zhijiang Yang, Tengxin Huang, Li Pan, Jingjing Wang, Liangliang Wang, Junjie Ding, Junhua Xiao

**Affiliations:** State Key Laboratory of NBC Protection for Civilian, Beijing, People’s Republic of China

**Correction: Journal of Cheminformatics (2024) 16:48** 10.1186/s13321-024-00843-y

Following publication of the original article [1], the authors identified a formatting error.

- The phrase "…with the computational cost exceeding 107 core-hours" in the **Abstract** should be replaced with "…with the computational cost exceeding 10^7^ core-hours"
- The statement "…with a total computational cost of more than 107 core-hours" in the **Conclusions** section should be changed to "…with a total computational cost of more than 10^7^ core-hours"

The original article has been corrected.
